# A feasibility trial of parent HPV vaccine reminders and phone-based motivational interviewing

**DOI:** 10.1186/s12889-020-10132-6

**Published:** 2021-01-09

**Authors:** Stephanie A. S. Staras, Eric Richardson, Lisa J. Merlo, Jiang Bian, Lindsay A. Thompson, Janice L. Krieger, Matthew J. Gurka, Ashley H. Sanders, Elizabeth A. Shenkman

**Affiliations:** 1 Department of Health Outcomes and Biomedical Informatics, College of Medicine, University of Florida, Gainesville, FL USA; 2 The Institute for Child Health Policy, College of Medicine, University of Florida, Gainesville, FL USA; 3 Department of Psychiatry, College of Medicine, University of Florida, Gainesville, FL USA; 4 Department of Pediatrics, College of Medicine, University of Florida, Gainesville, FL USA; 5 Department of Advertising, College of Journalism and Communication, University of Florida, Gainesville, FL USA

**Keywords:** Motivational interviewing, HPV vaccine, Text messaging, Feasibility, Acceptability

## Abstract

**Background:**

We assessed the feasibility and acceptability of a sequential approach of parent-targeted HPV vaccine reminders and phone-based Motivation Interviewing (MI).

**Methods:**

In 2016, we selected all 11- to 12-year-old boys and girls seen in one clinic whose vaccine records did not include the HPV vaccine (*n*=286). By gender, we individually randomized parents of adolescents to an interactive text message (74 girls and 45 boys), postcard reminder (46 boys and no girls because of previously demonstrated efficacy), or standard care group (75 girls and 46 boys). Reminders were sent with medical director permission and a HIPAA waiver. Two months after reminders, among the adolescents whose vaccine records still did not include the HPV vaccine, we selected a gender-stratified random sample of 20 parents for phone-based MI. We assessed the percentage of deliverable messages, the percentage of parents’ responding to the interactive text message, parent acceptability of receiving a text message, and MI parent responsiveness and interviewer competence (MI Treatment Integrity Coding system).

**Results:**

Nearly all messages were deliverable (98% of postcards and 74% of text messages). Six of the 88 parents (7%) receiving text messages scheduled an appointment through our interactive system. The acceptability survey response rate was 37% (38/102). Respondents were favorable toward vaccine reminders for all parents (82%). Among 20 sampled parents, 17 were reached by phone of whom 7 completed MI, 4 had or were getting the HPV vaccine for their child, and 5 expressed disinterest. Across the 7 MI calls, the interviewer was rated 100% MI adherent and scored an average 4.19 rating for Global Spirit.

**Conclusion:**

Without providing explicit consent to receive vaccine-related messages, parents nonetheless found postcards and interactive text messages acceptable. Centralizing MI to phone calls with trained staff was acceptable to parents and resulted in highly MI-adherent interviews.

**Supplementary Information:**

The online version contains supplementary material available at 10.1186/s12889-020-10132-6.

## Background

Despite the potential for the human papillomavirus (HPV) vaccine to prevent 31,200 cancer cases each year in the United States [1], only 73% of female and 70% of male 13- to 17-year-olds have initiated the HPV vaccine series (received of ≥ 1 dose) with some states having as few as 49% of adolescents initiating [2]. Likely the most effective way to increase HPV vaccination is for adolescents and their parents to receive clear and strong recommendations from their physician [3–7]. Within a well child visit, when vaccines are most commonly recommended and administered, the American Academy of Pediatrics recommends covering over 25 topics within the typically allotted 15 to 20 min [8–10]. Therefore, understandably, physicians report limited time to probe and discuss parental hesitations about the HPV vaccine [3, 11]. The time burden on physicians of addressing HPV vaccination could be eased with interventions that occur prior to and between clinic visits aimed at prompting clinic visits among acceptors, priming questioning parents with educational information, and addressing the specific concerns of more hesitant parents.

A common pre-clinic visit intervention for parents is sending vaccine reminders (e.g., autodialed phone calls, text-messages, or mailed postcards) that alert parents of recommended vaccines. Reminders are an effective and recommended strategy to increase vaccination including HPV vaccination [12–15]. Specifically for adolescent vaccination, however, reminders produce moderate increases in vaccination rates: a Cochrane review found only a 7% increase in vaccination rates from reminders across 10 studies with 30,868 participants [15–17]. Preliminary evidence suggests reminder effectiveness may be boosted with strategies like allowing parents to request a clinic call back, including educational information, and combining reminders with other interventions such as phone calls or navigator home visit [12, 13, 15, 18–21].

One well-established strategy to encourage individuals to adopt healthier behaviors, that to our knowledge, has not been applied in advance of clinic visit for vaccines, is Motivational Interviewing (MI) [22–27]. MI is a patient-centered, collaborative approach that uses specific skills (e.g. reflections, affirmations, open-ended questions) to enhance patients’ internal motivation to change by exploring resistance or ambivalence [28]. Several studies have taught physicians MI techniques as a way to improve communication with their patients regarding smoking, obesity, and even HPV vaccine hesitation [29–31]. The effectiveness of physician-delivered MI for vaccination as a component of primary care is limited by two primary challenges. First, the MI style conflicts with the tendency of many physicians to primarily give patients advice. Second, conducting MI proficiently is difficult, requires intensive training, and may require more time than a physician believes she or he can spend during a clinical visit [31–34]. However, there is evidence that MI interventions can be conducted successfully by a non-physician outside the typical clinic visit. For example, to increase the reach of an MI intervention for substance use disorder, MI was performed over the telephone by trained clinicians outside of an office-based visit [35].

Before the effectiveness of a pre-clinic intervention can be tested in a larger randomized control trial, an evaluation of fit must be conducted. According to Proctor et al., feasibility and acceptability are two of the three most important ways of determining how well an intervention fits within a particular setting [36]. Following best practices for conducting pilot studies [37], we evaluated the acceptability and feasibility of a two-phased, parent-targeted intervention to increase HPV vaccine initiation among 11- to 12-year-olds between May and November 2016. In the first phase, we sent reminders to all parents attending a clinic by capitalizing on the authority of medical clinics to inform parents of recommended services. The reminder (postcard or text message) included educational information about the HPV vaccine identified as most relevant to parents in North Central Florida – cancer prevention and safety [5]. In the text messages, we streamlined the call-to-action by including an interactive scheduling system. In the second phase, a sample of parents who did not have their child receive the HPV vaccine following the reminders were invited to phone-based MI sessions with a trained psychologist. We evaluated acceptability and feasibility regarding (1) delivery of reminder messages, (2) parent acceptability of reminders and phone-based MI, (3) interviewer MI proficiency.

## Methods

### Study population

Using one North-Central Florida, academic, primary care clinic’s electronic health record (EHR) data, we identified all 11- to 12-year-old girls and boys (date of birth 7/2/2003 to 4/1/2005) who had visited the clinic in the preceding year (3/1/2015 to 2/29/2016). Among the 548 adolescents, we selected the 325 adolescents whose immunization history did not include the HPV vaccine as of April 14, 2016 in the clinic’s EHR billing and immunization history: HPV quadrivalent vaccine Gardasil, HPV-9 valent vaccine Gardasil 9, or HPV bivalent vaccine Cervarix. To find additional vaccine records, we matched the adolescents to the enrollment records for the Florida Children’s Health Insurance Program (CHIP) and Medicaid Programs of Managed Care and Fee for Service using two algorithms: (1) date of birth, last name, and first four letters of first name or (2) date of birth, first two letters of last name, and city of residence. With this strategy, we matched 280 records including 97% of the 234 listed as Medicaid insured in the EHR. We eliminated the 39 adolescents who had Medicaid or CHIP claims for the HPV vaccine (Current Procedural Terminology Codes 90,649, 90,650, and 90,651) reported by May 4th, 2016. The final study population was 286 adolescents (137 boys and 149 girls).

### Study design

**Fig. 1:**
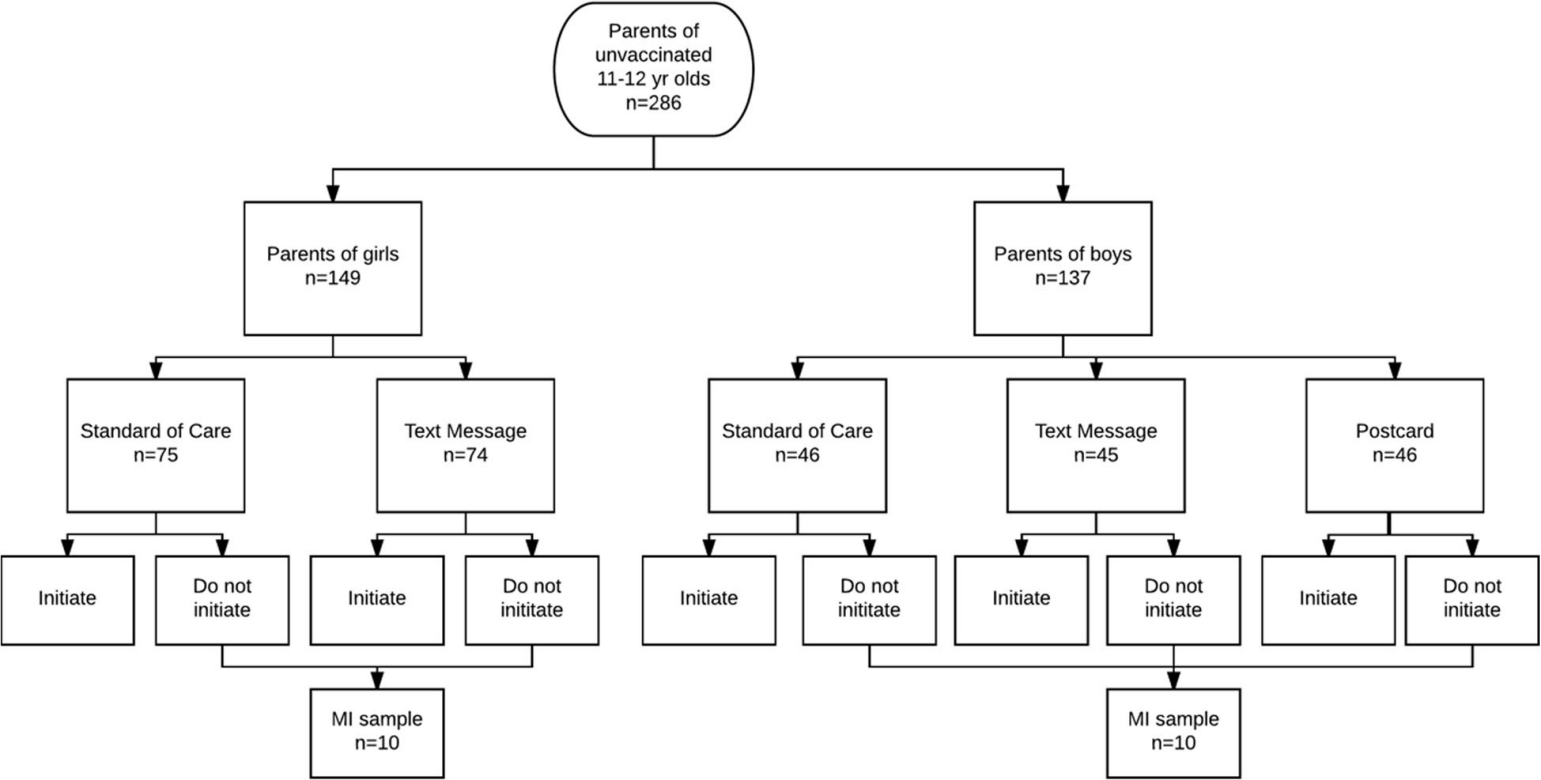
Multi-armed factorial randomized study design

We used a two-phased, factorial design with simple randomization to assign parents to interventions (Fig. 1). Parents of boys were equally randomized into one of three conditions: postcard (*n*=46), text messaging (*n*=45), and standard of care (*n*=45). Due to our previous study demonstrating the efficacy of postcards among girls [38], we randomized parents of girls to two conditions: text messaging (*n*=74) and standard of care (*n*=75). To test the feasibility of a one-time, phone-based MI session among parents non-responsive to reminders, we selected a gender-stratified random sample of 20 adolescents who did not have HPV vaccine records by 60 days after the reminder (July 11, 2016) that were available in the EHR by July 29, 2016 or in Medicaid claims by August 9, 2016.

### Intervention components

#### Postcard and text reminders

To send the messages without acquiring explicit permission from the parents, we sent our postcard and text-message reminders under the authority of the medical director of the primary care clinic and obtained a Health Insurance Portability and Accountability Act waiver of authorization. Moreover, messages were applicable to all 11- to 12-year-olds and did not reference the child’s vaccine record.

Based on our previous effective postcards among girls [38], our postcard and text messages included key words (“protect,” “safe,” and “free”), were sent in English and Spanish, and were personalized to include the first name of the child in the message. Based on our previous postcard strategy [38], we revised our boys’ postcard to include a picture of an African American mother and son and the phrases “Protect Your Son from Cancer” and “Get the Facts.” The six-by-eight inch, full-color postcards included a web address and phone number for more information. For each boy, we obtained addresses from the EHR (primary) or Medicaid enrollment (secondary). Prior to mailing, we updated addresses with the National Change of Address Database. On May 18, 2016, we sent one postcard to the boys’ parents or guardians via first class mail with return service requested.

Also, on May 18, 2016, we sent text messages to the phone number in the EHR (primary) or Medicaid enrollment (secondary) from the same phone number and vendor as the clinic’s appointment reminder system. Text messages said: “Did you know you can protect [insert child’s first name] from certain cancers? The HPV vaccine is safe, free, and easy. Reply Yes to have [insert child’s first name]’s doctor’s office call you.” If the parent replied “Yes,” they received a reply stating that the clinic staff would call within two business days to schedule an appointment. Clinic schedulers called text responders. We looked for and sent text messages to alternative phone numbers when initial text messages were non-deliverable.

#### MI phone calls

On August 22, 2016, we mailed invitation letters to inform the parents of the 20 selected adolescents of the upcoming MI calls. Between August 25, 2016 and September 13, 2016, we attempted to call parents at the phone number in the EHR at various times of the day, for up to ten calls per number. A licensed clinical psychologist and member of the Motivational Interviewing Network of Trainers (LM) conducted and audio recorded all phone calls. Following parent consent, the psychologist guided the parent in a conversation designed to help them make a fully-informed decision about vaccinating their child for HPV with open-ended questions, reflective listening, and a focus on individual concerns and autonomy support [23, 24, 39]. An approximate conversation guide led the interviewer through establishing a relationship with the parent in a general discussion about vaccines, providing HPV vaccine information, answering questions, and aiding the parent in setting a personal goal regarding the HPV vaccine for their child, if appropriate (see Supplement A). The study team provided the psychologist with information to address commonly asked questions about the HPV vaccine. Mobile phone minute fee reimbursement was available for participants upon request.

### Feasibility and acceptability measures

#### Reminders

We assessed the acceptability and feasibility of reminders in four ways. First, we assessed whether messages were delivered (non-returned postcards or confirmed deliverable text messages). Second, we assessed parents’ response to messages by tracking the number of requests that messages stop, calls to the phone number provided on the postcard for more information, and unique hits to the website provided on the postcard. Third, we assessed the interactive scheduling component by tracking the percentage of parents that responded yes and the outcomes of returned calls. For each responding parent, clinic staff tracked contact attempts, scheduled appointments, and HPV-related questions asked. Fourth, we sent a brief one-page acceptability survey to up to 50 randomly-selected parents within each of three groups (text message responders, text message non-responders, and postcard recipients). Questions included parents’ recall of the reminder, opinions about the reminder, actions taken upon receiving the reminder, and expectations and experiences with interactive scheduling (if applicable) (see supplement B). We sent each parent selected for the survey a notification postcard (June 14, 2016), a survey with a $10 cash incentive via FedEx (June 23, 2016), and randomized non-responders equally to an additional mailed survey or a texted online survey link (July 28, 2016).

#### MI

We assessed phone-based MI acceptability and feasibility by parent participation rates and behavioral coding of transcribed interviews. A trained MI coder independent from the study team used the MI Treatment Integrity (MITI) Coding manual 3.1 to assign each interview a score for global ratings and MI techniques [23, 24, 39]. Particularly, in pilot studies, using one MI coder is typical for MITI scoring [40–43]. The global ratings capture the holistic performance of the interviewer and reflect evocation, collaboration, autonomy/support, direction and empathy. Each global rating is coded on a scale of 1 to 5 (1=low and 5=high). Additionally, a global spirit score is the calculated average of the global ratings for evocation, collaboration and autonomy/support [44]. A global spirit score of 3.5 is considered beginner proficiency and ≥4 is considered to be competent. MI techniques are measured with counts of instances of an interviewer’s behavior for important skills: giving information (e.g. provide feedback, educational material or explaining an idea), questions (open vs. closed questions), reflections (simple vs. complex), and MI adherence/MI non-adherence. Summary scores are created for each category by calculating ratios of ideal versus overall behavior (e.g. the percentage of questions that are open-ended). We used the proficiency and competency cutoff scores recommended in the MITI 3.0 as benchmarks.

### Statistical analysis

Because this was a pilot study, we focused analysis on descriptive statistics and frequencies of the process measures and MITI scores. Following the CONSORT guidelines for pilot and feasibility studies [37], we did not perform any formal hypothesis testing for effectiveness. Analysis was conducted with SAS 9.4 (SAS Institute Inc., Cary, NC).

## Results

### Feasibility of HPV vaccine reminders

The majority of messages were delivered and acceptable to parents. Postcards were delivered to 98% (45/46) of parents of boys. We confirmed delivery of text messages to the listed phone numbers for 80% (36/45) of parents of boys and 70% (52/74) of parents of girls. Among parents for whom text messages were confirmed deliverable, 8% (*n*=7) requested messages stop. One parent called the number on the postcard to ask where they could get the HPV vaccine. Within the 6 months following the messaging, we had 213 unique visits to the website listed on the postcard. Most visitors only saw the home page, but each of our six topical links received 30 to 40 views.

For the interactive scheduling component, 7% (*n*=6) of parents for whom we confirmed delivery of the text message replied that they would like the clinic to call to schedule an appointment. All were called within 2 business days of replying to the text and 83% (5/6) were reached on the first attempt. All responding parents scheduled an appointment and 66% (4/6) scheduled an appointment the day the text message was sent.

### Acceptability of HPV vaccine reminders

We sent an acceptability survey to the 45 parents who received our postcard, the six parents who responded to the text message, and a random sample of 50 parents who did not respond to deliverable text messages. The overall survey response rate was 37% (38 /102) with returned completed surveys from 19 parents who received postcards, one parent who responded to the text message, and 18 parents who did not respond to a deliverable text message. The vast majority (82%) of the respondents agreed that all parents should receive reminders about the HPV vaccine (Table 1). Among the 55% (21/38) of parents who reported remembering receiving either a text or postcard message, 100% agreed that the reminders were easy to understand and that they trusted the information provided. A majority of parents liked receiving the reminders (80%). Importantly, over half of the parents (62%) reported taking some action after receiving the reminders, such as calling their doctor, speaking to their child, or speaking to a friend or family member. The majority of parents recalled that the reminders included information on the HPV vaccine benefits (86%) and safety (76%).

**Table 1:** Acceptability of messages among 38 responding parents

	Postcard N (%)	Text Message N (%)	Total N (%)
Total	19	19	38
All parents should receive a reminder	15 (79%)	16 (84%)	31 (82%)
Remembered receiving message	12 (63%)	9 (47%)	21 (55%)
Reminders were easy to understand	12 (100%)	9 (100%)	21 (100%)
Trusted information on reminders	12 (100%)	9 (100%)	21 (100%)
Liked receiving the reminders	9 (75%)	8 (89%)	17 (81%)
Reaction to message			
Any conversations or information seeking	7 (63%)	6 (67%)	13 (62%)
Spoke with your child about the HPV vaccine	2 (17%)	3 (33%)	5 (24%)
Looked up the HPV vaccine on the Internet	3 (25%)	3 (33%)	6 (29%)
Spoke with friends or family or read about it	2 (17%)	0 (0%)	2 (10%)
Believed message told about HPV vaccine			
Benefits	11 (92%)	7 (77%)	18 (86%)
Costs	6 (50%)	6 (67%)	12 (57%)
Safety	10 (83%)	6 (67%)	16 (76%)

### Feasibility and acceptability of phone-based motivational interviewing

Among the 20 randomly selected parents, the interviewer was able to reach 17 (85%). This required an average of 4 attempts (range = 1–10). MI was completed with 7 parents (35%). Among the 10 parents who were contacted but did not participate in MI, four reported they had already had their child receive or were trying to get the HPV vaccine, three expressed general disinterest, one expressed disinterest in HPV specifically, one hung up during the description, and one said to call back, but never answered again. MI calls lasted between 3 to 27 min (average length = 10:47 min). During the goal setting portion of the seven MI sessions, one parent reported she already had her child receive the HPV vaccine, one parent described her opposition to all vaccines, one parent wanted to conduct additional research before deciding, and four set a goal to get the HPV vaccine for their child during his or her next preventive visit. None of the contacted parents complained about the call.

### Proficiency of phone-based motivational interviewing

Across calls, the interviewer competently followed the principles of MI indicated by an average Global Spirit Rating above 4 (score = 4.19, standard deviation = 0.50) (Table 2). The interviewer displayed proficiency for each global rating score with an average over 3.5. Scores for four of the five global metrics were above 4 demonstrating competency. The interviewer scored a 5 on direction indicating the interviewer focused the conversation on the HPV vaccine and selectively reinforced discussion about the HPV vaccine. Using open questions for 56% of the questions, she showed interviewer proficiency (> 50%). The interviewer scored below proficiency for percentage of complex reflections and the reflection to question ratio. Upon review, it became apparent that a number of reflections were “spoiled” by intonation, resulting in them being coded as closed-ended questions. The interviewer performed competently for adhering to MI principles 100% of the time.

**Table 2:** MITI codes for seven MI phone calls regarding HPV vaccination

	Average	Standard Deviation	Proficiency Score	Competency Score
Length (minutes: seconds)	10:47	8:15		
Global Spirit rating (1 to 5)	4.19	0.50	≥ 3.5	≥4
Global Ratings (1 to 5)				
Evocation	3.86	0.69	≥ 3.5	≥4
Collaboration	4.43	0.53	≥3.5	≥4
Autonomy /Support	4.29	0.49	≥3.5	≥4
Direction	5	0	≥3.5	≥4
Empathy	4.14	0.38	≥3.5	≥4
MI Techniques				
Giving Information	2.29	2.06	N/A	N/A
Percent of Complex Reflections	25%	21%	≥40%	≥50%
Percent Open Questions	56%	14%	> 50%	> 70%
Reflection to Question Ratio	0.68	0.17	≥1	≥2
% MI Adherent	100%	0	≥90%	100%

## Discussion

Among parents of 11- to 12-year-olds attending a primary care clinic, we demonstrated the feasibility and acceptability of our sequential approach of parent-targeted HPV vaccine reminder messages. Our phone-based MI was acceptable to parents and feasible to conduct with a MINT trained professional. By and large, parents received and responded positively to vaccine reminders that they had not explicitly consented to receive. Nearly one out of ten parents scheduled clinical appointments by using our interactive text message system. Phone-based MI was conducted without complaints and performed competently with participating parents. A larger trial is warranted to test the effectiveness of the conjunctive use of text messaging and phone-based MI as pre-visit interventions to boost HPV vaccination rates without placing additional burden on physicians.

Despite not consenting to receive reminders, parents did not seem to find the contacts from the research team objectionable. We did not receive any complaints, and we received few opt-out requests from parents. While it is common that explicit consent is not collected for reminders for other vaccines, many HPV vaccine reminder studies have used explicit parent consent to receive vaccine reminders [17, 18, 45, 46]. Thus, many interventions on HPV vaccine are limited to addressing HPV vaccine completion. By demonstrating the acceptability of parent reminders without explicit consent in a general pediatric setting in the southern United States, future trials can test the effectiveness of personalized messages to increase HPV vaccine initiation. Additionally, consistent with parents’ endorsement of text reminders for infant vaccines [47], the 37% of parents who responded to our survey were overwhelmingly positive about the personalized reminders. The low response rate to the acceptability survey leaves room for potential non-response bias and potentially reduces generalizability to our target population. Our response rates, however, are comparable to other studies with parents of adolescents (38 to 48%) [38, 48–51].

Based on the parents that scheduled appointments, interactive scheduling is a feasible strategy both for the parents and clinic. The clinic was able to call responding parents within 2 days and reached most parents on the first attempt. Likewise, the follow through for scheduling appointments when called suggests interactive text messages can be a prompt to help parents enact a plan [52]. Thus, interactive scheduling likely meets the needs of the estimated 10% of parents who intend to vaccinate their child in the next year, but have not gotten around to it [53]. Our rates of parents requesting a clinic call back in response to the text message (7%) was comparable to a previous study that allowed parents to respond to text messages indicating needed vaccines (10%) [18]. For a larger trial, our pilot strategy using interactive scheduling might consider enhancements such as timing text messages more closely to scheduled visits or milestones, completing the scheduling process immediately via text, or targeting optimal times during the week and day to send reminders.

Phone-based MI using phone numbers from EHR or claims records was acceptable according to parents and feasibly conducted by a trained MINT provider. Our agreement rate of 35% is comparable with other studies of telephone-based MI that ranged from 26 to 50% agreement and can be used to help plan sample sizes for future trials [35, 54]. When compared with physicians trained to perform MI during primary care, our telephone-based approach allowed the use of a highly trained MI provider to conduct the sessions and resulted in the precepts of MI being closely followed. For example, all sessions in our study were highly MI compliant, whereas, in a study of physicians trained to use MI to discuss HPV vaccination, only 28% of physicians reported using affirmations or reflections more than 75% of the time [31]. Even highly motivated physicians trained during 6 MI workshops found beginning proficiency for MI difficult to achieve [55]. Additionally, phone-based MI allowed parents more time than likely available during a well child visit to discuss concerns: our average MI conversation of 10 min may be difficult to achieve within a 15 to 20 min well visit [8].

This study has three important limitations. Because it was a feasibility study, we had a small sample: one clinic, one interviewer, and 20 attempted MI interviews. The low sample size prevented us from making meaningful conclusions about effects on vaccination. Future trials can include a larger sample to assess efficacy of the interventions. Second, we did not collect information on what happened during clinic visits (e.g., parent mentioning of reminders or phone calls, physician recommendations, and time spent discussing the vaccine). Thus, we were unable to assess any intermediate effects or potential moderators of the interventions. Third, while we measured delivery of messages, we were unable to assess if messages or phone calls among those who never answered went to the correct person.

This study has three main strengths. First, we used MI as a strategy to reduce parents’ HPV vaccine hesitancy and measured interviewer competency with a standardized tool, the MITI. This combination, particularly with phone-based delivery, is novel. Second, we measured feasibility of reminders with a variety of process measures including messages delivered, website hits, and a parent acceptability survey. Third, medical and administrative Medicaid and CHIP records provided reasonably accurate contact information for the parents of 11- to 12-year-old clinic patients.

## Conclusions

Pre-appointment interventions, such as reminder messages and phone-based MI, are feasible strategies to respond to the President’s Cancer Panel’s goal of increasing parents’ acceptance of the HPV vaccine. The positive responses by parents who completed the acceptability survey, the number of visitors to our website, and the in-depth conversations during MI phone calls suggest that the interventions addressed at least some of the needs of parents. Phone-based MI may be a feasible alternative to address the limitations of training physicians in MI as way to reach the parents hesitant to the HPV vaccine. A larger effectiveness trial is warranted and should consider shortening the time between interventions and clinic visits. While evidence suggests that reminder messages are cost-effective [56], future trials should evaluate if the addition of phone-based MI achieves sufficient increases in vaccination to justify the additional expense. Expanding upon the priming effects seen with pre-clinic vaccine reminders [57], if shown effective, more intense pre-clinic interventions like phone-based MI may reduce physician time needed to address parental HPV vaccine hesitancy.

## Supplementary Information

**Additional file 1.**

**Additional file 2.**

## Data Availability

The data that support the findings of this study are available on request from the corresponding author [SS] with a signed data use agreement. The data are not publicly available due to the small sample from one clinic potentially compromising the research participant privacy.

## Abbreviations

CHIP: Children’s Health Insurance Program
HPV: Human papillomavirus
MI: Motivational Interviewing
