# Supplementary material for: A feasibility trial of parent HPV vaccine reminders and phone-based motivational interviewing
Source: BMC Public Health. 2021 Jan 9;21:109. doi: 10.1186/s12889-020-10132-6 (PMC7797089; doi:10.1186/s12889-020-10132-6)
Supplement: Supplementary file 1 — Additional file 1. [file 12889_2020_10132_MOESM1_ESM.docx]

Motivational Interviewing

HPV vaccination

This document includes to components to help guide the interview: a conversation guide and a list of relevant HPV vaccination topics.

**A. Conversation Guide**

1. Establish a relationship

Begin with a general question regarding their child’s health and how they as parents feel about recommended health practices for their adolescent children. For example, **“I’m curious—how comfortable are you with the recommended health measures like vaccines for {child’s name}?”**

- Follow up using basic MI skills: affirmations, reflections, open-ended questions. The overall goal here is to gain trust with the parents and to demonstrate your *genuine* interest to understand their situation and opinions.
- This section should take no more than 5-8 minutes

1. Provide information

If it is OK with the participant, mention that you have some information you would like to share. **“I have some important information about how vaccines for 11-12 year olds, like {child’s name}, can help prevent diseases like cancer. Is it OK with you if we spend 1 or 2 minutes talking about them?”**

- Participant says yes
  - Provide the participant with general information about HPV vaccine.
  - Follow up with the respondent employing the same basic MI skills: affirmations, reflections, and open-ended questions. Try to elicit change talk by asking for explanations and being genuinely curious about the participant’s opinions. Try asking them non-threatening questions, for example: **“What do you think of all the information I just shared?”**
- Participant says no
  - Be completely objective and remember you are not evaluating the participant. Follow up with the respondent employing the same basic MI skills: affirmations, reflections, and open ended questions. Try to elicit change talk by asking for explanations and being genuinely curious about the participant’s opinions. For example, **“Not a problem. So, if I may ask, what is your reaction to what we have discussed so far?”**

1. Closing

- With 5-8 minutes left in the session summarize what you have discussed so far. **“Let me see if I understand where you are at with your child’s immunizations and health right now . . .”**
  - Summarize the positive health behaviors the participant feels good about already, start generally and then move into specifics (be sure to include the HPV vaccine if applicable).
  - After summarizing the positive health behaviors move on to any subjects of concern that the participant brought up. Again start generally and move onto specifics (be sure to include the HPV vaccine if applicable).
  - Allow the participant to make his or her own conclusions based on the conversation and encourage them to take action on their positive health behaviors (example: talk to your doctor, schedule an appointment, talk about the HPV vaccine with your child, etc.)

**B. HPV Vaccination Topics**

Concerns with vaccination may be related to vaccine or they may be more practical concerns about getting to the clinic with their child. More practical concerns can be addressed by helping parents identify their options – busses, taxi, babysitters for younger children, times when they are available, etc.

**1)** How do I know the HPV vaccine is safe for my child?

The HPV vaccination is safe. Doctors and other health care specialists are comfortable saying this because of how the vaccine is made, how many people have received the vaccine already, and the lack of serious side effects.

How is the HPV vaccine made?

- The HPV vaccine is made using only the surface protein from the human papillomavirus, which means the HPV vaccine can’t cause cancer, but your child’s immune system can quickly recognize and destroy any HPV virus before it causes an infection.


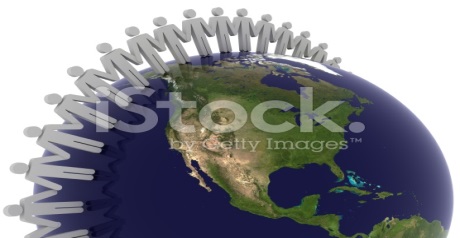
How new is the vaccine?

- The HPV vaccine became available a decade ago in 2006. Since then the HPV vaccine has been routinely given to over 67 million people. If all 67 million people came together to make one line they could circle the globe twice!

Mild and short-term side effects:

- Nearly all (99%) of teens who got the vaccine did not have any side effects. On rare occasions teens experienced mild and short-term side effects right after getting the vaccine, these include:
  - - Redness and tenderness in the arm where the shot was given
    - Headache
    - Low grade fever
    - Nausea
    - Feeling tired
    - Fainting

No evidence of serious side effects

- No serious side effects have ever been linked to the HPV vaccine. The HPV vaccine is monitored continuously by the Food and Drug Administration (FDA), the Centers for Disease Control and Prevention (CDC), and other independent organizations.
- You may have seen or heard of reports of serious side effects, but none of those cases have turned out to be caused by the vaccine.

**2)** How do I know if my child is at risk for getting HPV or related conditions?

HPV infections are very common. About 79 million Americans are currently infected with HPV. About 14 million people become newly infected each year. HPV is so common that most sexually-active men and women will get at least one type of HPV at some point in their lives.

**3)** HPV infections cause cancer

Every year in the United States, 27,000 people get cancers caused by HPV infection. That’s 1 person every 20 minutes of every day all year long!

HPV can cause cervical and other cancers including cancer of the vulva, vagina, penis, or anus. It can also cause cancer in the back of the throat, including the base of the tongue and tonsils (called [oropharyngeal cancer](http://www.cdc.gov/cancer/hpv/statistics/headneck.htm)).

**4)** My child is still too young, maybe next year . . .

The HPV vaccine works best if given to preteen boys and girls at age 11 - 12 because that’s the time when their bodies will produce the right amount of infection-fighting cells, also known as antibodies. By giving your child the vaccine at the recommended time you are giving their body time to prepare well before they are exposed to the virus.

The point of vaccination is to make sure your child has protection long before they are exposed to the virus.

**5)** Do both girls and boys need the HPV vaccine?

Yes! The vaccine is recommended for boys and girls to protect them from getting cancer.

**6)** How much does the HPV vaccine cost?

Vaccination is likely free of charge. There may be small administrative costs depending on your insurance.
