# Supplementary material for: A feasibility trial of parent HPV vaccine reminders and phone-based motivational interviewing
Source: BMC Public Health. 2021 Jan 9;21:109. doi: 10.1186/s12889-020-10132-6 (PMC7797089; doi:10.1186/s12889-020-10132-6)
Supplement: Supplementary file 2 — Additional file 2. [file 12889_2020_10132_MOESM2_ESM.docx]

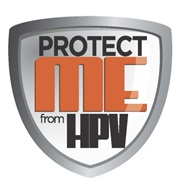


**Did our HPV vaccine text message help you?**

A few weeks ago, we sent you a text message about the human papillomavirus (HPV) vaccine. We would like to know what you thought about the text message. We would like the parent or guardian of **{child’s first name}** to complete this survey. Your participation is very important. Please return the survey in the postage-paid envelope provided by **{date will be inserted}.**

*The human papillomavirus (HPV) is a common virus known to cause genital warts and some cancers. A vaccine to prevent HPV infection is available and is called the cervical cancer vaccine, genital warts vaccine, HPV shot, GARDASIL, GARDASIL 9, or CERVARIX*

**1. Did you do any of the following after you received the text message? (*Mark all that apply)***

□ Speak with your child about the HPV vaccine

□ Speak with friends or family (other than your children) about the HPV vaccine

□ Look up the HPV vaccine on the Internet

□ Make an appointment to talk to your child’s doctor about the HPV vaccine

□ Reply Y

□ Other, please write here __________________

**2. When you replied Y, did you expect {child’s name}’s doctor’s office to call and (*Mark all that apply)***

□ Answer your HPV vaccine questions

□ Schedule an appointment with {child’s name}’s doctor

□ Schedule an appointment to get the vaccine from a nurse

□ Other, please write here ____________________________

**3. When {child’s name}’s doctor’s office called you how satisfied were you with the following?**

|  | Extremely satisfied | Satisfied | Neither satisfied nor dissatisfied | Dissatisfied | Extremely dissatisfied |
| --- | --- | --- | --- | --- | --- |
| How quickly they called | □ | □ | □ | □ | □ |
| The information they gave you | □ | □ | □ | □ | □ |
| The overall experience | □ | □ | □ | □ | □ |

**4. How strongly do you agree with each of the following statements about the text message?**

|  | Strongly Agree | Agree | Neither Agree nor Disagree | Disagree | Strongly Disagree | Don’t Know |
| --- | --- | --- | --- | --- | --- | --- |
| It was easy to understand | □ | □ | □ | □ | □ | □ |
| I trusted the information | □ | □ | □ | □ | □ | □ |
| It told me about the benefits of the HPV vaccine | □ | □ | □ | □ | □ | □ |
| It told me about the safety of the HPV vaccine | □ | □ | □ | □ | □ | □ |
| It told me about the cost of the HPV vaccine | □ | □ | □ | □ | □ | □ |
| I liked receiving it | □ | □ | □ | □ | □ | □ |

**5. Do you recommend all parents of 11-12 year olds receive a text message reminder about the HPV vaccine?**

□ Yes

□ No

□ Don’t know

**6. How would you like to receive additional health-related information about the HPV vaccine for {child’s name}? (Mark all that apply)**

□ By postcard

□ By text message

□ By mailed informational brochure

□ By internet website

□ By social media (e.g., Facebook, Twitter, and Instagram)

□ By a phone call with a health professional

□ I would NOT like to receive health-related information

**7. Do you plan to get the HPV vaccine for {Child’s name}?**

□ Yes

□ No

□ I have not decided

□ I have not thought about it

□ {Child’s name} already received the vaccine

**8. What additional information would help you decide about getting the HPV vaccine for {child’s name}? (Mark all that apply)**

□ I have already decided about the HPV vaccine

OR

.

□ If the vaccine works □ Advantages of getting the vaccine at 11-12 years vs. later

□ Cost of the vaccine □ Learning more about the human papillomavirus (HPV)

□ Pain of the shots □ Whether teens who get the vaccine are more likely to have sex

□ Vaccine safety □ Whether my friends and family got the vaccine for their children

□ My child’s risk of cancer □ Whether my doctor thinks my child should receive the vaccine

□ Other, please write here __________________

**9. What would motivate you to get the HPV vaccine for {Child’s name} in the next two weeks? (Mark all that apply)**

□ Child already has all vaccine doses or is not due for the next dose

□ $25 gift card

□ Voucher for transportation to {child’s name}’s doctor

□ Additional information about the vaccine

□ Discussion of my concerns with a health professional

□ Other, please write here _______________________

□ Nothing, I will not get the HPV vaccine for {Child’s name}

**Thank you for completing this survey! Please return it in the envelope provided.**


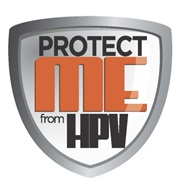


**Did our HPV vaccine text message help you?**

A few weeks ago, we sent you a text message about the human papillomavirus (HPV) vaccine. We would like to know if you received the text message and what you thought about the text message. We would like the parent or guardian of **{child’s first name}** to complete this survey. Your participation is very important.

Please return the survey in the postage-paid envelope provided by **{date will be inserted}.**

*The human papillomavirus (HPV) is a common virus known to cause genital warts and some cancers. A vaccine to prevent HPV infection is available and is called the cervical cancer vaccine, genital warts vaccine, HPV shot, GARDASIL, GARDASIL 9, or CERVARIX*

1. **Did you receive a text message about the HPV vaccine?**

□ Yes

□ No (Skip to Question 5)

□ Don’t know (Skip to Question 5)

**2. Did you do any of the following after you received the text message?(*Mark all that apply)***

□ Speak with your child about the HPV vaccine

□ Speak with friends or family (other than your children) about the HPV vaccine

□ Look up the HPV vaccine on the Internet

□ Make an appointment to talk to your child’s doctor about the HPV vaccine

□ Nothing

□ Other, please write here __________________

**3. Why did you choose not to respond to the text message? (*Mark all that apply)***

□ I called {child’s name} doctor myself

□ I forgot

□ I didn’t know that I could respond to the message

□ My child already had an appointment with his or her doctor

□ {Child’s first name} already had the HPV vaccine

□ I was not interested in the HPV vaccine for {child’s first name}

□ I was annoyed at getting the text message

□ Other, please write here __________________

**4. How strongly do you agree with each of the following statements about the HPV text message?**

|  | Strongly Agree | Agree | Neither Agree nor Disagree | Disagree | Strongly Disagree | Don’t Know |
| --- | --- | --- | --- | --- | --- | --- |
| It was easy to understand | □ | □ | □ | □ | □ | □ |
| I trusted the information | □ | □ | □ | □ | □ | □ |
| It told me about the benefits of the HPV vaccine | □ | □ | □ | □ | □ | □ |
| It told me about the safety of the HPV vaccine | □ | □ | □ | □ | □ | □ |
| It told me about the cost of the HPV vaccine | □ | □ | □ | □ | □ | □ |
| I liked receiving it | □ | □ | □ | □ | □ | □ |

**5. Do you recommend all parents of 11-12 year olds receive a text message reminder about the HPV vaccine?**

□ Yes

□ No

□ Don’t know

**6. How would you like to receive additional health-related information about the HPV vaccine for {child’s name}? (Mark all that apply)**

□ By postcard

□ By text message

□ By mailed informational brochure

□ By internet website

□ By social media (e.g., Facebook, Twitter, and Instagram)

□ By a phone call with a health professional

□ I would NOT like to receive health-related information

**7. Do you plan to get the HPV vaccine for {Child’s name}?**

□ Yes

□ No

□ I have not decided

□ I have not thought about it

□ {Child’s name} already received the vaccine

**8. What additional information would help you decide about getting the HPV vaccine for {child’s name}? (Mark all that apply)**

□ I have already decided about the HPV vaccine

OR

.

□ If the vaccine works □ Advantages of getting the vaccine at 11-12 years vs. later

□ Cost of the vaccine □ Learning more about the human papillomavirus (HPV)

□ Pain of the shots □ Whether teens who get the vaccine are more likely to have sex

□ Vaccine safety □ Whether my friends and family got the vaccine for their children

□ My child’s risk of cancer □ Whether my doctor thinks my child should receive the vaccine

□ Other, please write here __________________

**9. What would motivate you to get the HPV vaccine for {Child’s name} in the next two weeks? (Mark all that apply)**

□ Child already has all vaccine doses or is not due for the next dose

□ $25 gift card

□ Voucher for transportation to {child’s name}’s doctor

□ Additional information about the vaccine

□ Discussion of my concerns with a health professional

□ Other, please write here _______________________

□ Nothing, I will not get the HPV vaccine for {Child’s name}

**Thank you for completing this survey! Please return it in the envelope provided.**


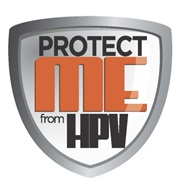


**Did our HPV vaccine postcard help you?**

A few weeks ago, we sent you a postcard about the Human Papillomavirus (HPV) vaccine. We would like to know if you received the postcard and what you thought about the postcard. We would like the parent or guardian of **{child’s first name}** to complete this survey. Your participation is very important.

Please return the survey in the postage-paid envelope provided by [**insert** **date**].

*The human papillomavirus (HPV) is a common virus known to cause genital warts and some cancers. A vaccine to prevent HPV infection is available and is called the cervical cancer vaccine, genital warts vaccine, HPV shot, GARDASIL, GARDASIL 9, or CERVARIX.*

1. **Did you receive a postcard about the HPV vaccine?**

□ Yes

□ No (Skip to question 4)

□ Don’t know (Skip to question 4)

**2. Did you do any of the following after you received the postcard? (*Mark all that apply)***

□ Speak with your child about the HPV vaccine

□ Speak with friends or family (other than your children) about the HPV vaccine

□ Look up the HPV vaccine on the internet

□ Go to the website provided on the postcard ([www.protectmefromhpv.com](http://www.protectmefromhpv.com))

□ Call the telephone number on the postcard

□ Make an appointment to talk to your child’s doctor about the HPV vaccine

□ Nothing

□ Other, please write here __________________

**3. How strongly do you agree with each of the following statements about the postcard?**

|  | Strongly Agree | Agree | Neither Agree nor Disagree | Disagree | Strongly Disagree | Don’t Know |
| --- | --- | --- | --- | --- | --- | --- |
| It was easy to understand | □ | □ | □ | □ | □ | □ |
| I trusted the information | □ | □ | □ | □ | □ | □ |
| I liked the colors | □ | □ | □ | □ | □ | □ |
| I liked the pictures | □ | □ | □ | □ | □ | □ |
| It told me about the benefits of the HPV vaccine | □ | □ | □ | □ | □ | □ |
| It told me about the safety of the HPV vaccine | □ | □ | □ | □ | □ | □ |
| It told me about the cost of the HPV vaccine | □ | □ | □ | □ | □ | □ |
| I liked receiving it | □ | □ | □ | □ | □ | □ |

**4. Do you recommend all parents of 11-12 year olds receive a postcard reminder about the HPV vaccine?**

□ Yes

□ No

□ Don’t know

**5. How would you like to receive additional health-related information about the HPV vaccine for {child’s name}? (mark all that apply)**

□ By postcard

□ By text message

□ By mailed informational brochure

□ By Internet website

□ By social media (e.g., Facebook, Twitter, and Instagram)

□ By a phone call with a health professional

□ I would NOT like to receive health-related information

**6. Do you plan to get the HPV vaccine for {Child’s name}?**

□ Yes

□ No

□ I have not decided

□ I have not thought about it

□ {Child’s name} already received the vaccine

**7. What additional information would help you decide about getting the HPV vaccine for {child’s name}? (Mark all that apply)**

□ I have already decided about the HPV vaccine

OR

.

□ If the vaccine works □ Advantages of getting the vaccine at 11-12 years vs. later

□ Cost of the vaccine □ Learning more about human papillomavirus (HPV)

□ Pain of the shots □ Whether teens who get the vaccine are more likely to have sex

□ Vaccine safety □ Whether my friends and family got the vaccine for their children

□ My child’s risk of cancer □ Whether my doctor thinks my child should receive the vaccine

□ Other, please write here __________________

**8. What would motivate you to get the HPV vaccine for {Child’s name} in the next two weeks? (Mark all that apply)**

□ Child already has all vaccine doses or is not due for the next dose

□ $25 gift card

□ Voucher for transportation to {child’s name}’s doctor

□ Additional information about the vaccine

□ Discussion of my concerns with a health professional

□ Other, please write here _______________________

□ Nothing, I will not get the HPV vaccine for {Child’s name}

**Thank you for completing this survey! Please return it in the envelope provided.**
